# 411 Mrd. €, 11,9 %

**DOI:** 10.1007/s00103-022-03539-6

**Published:** 2022-05-25

**Authors:** Joseph Kuhn

**Affiliations:** Bayerisches Landesamt für Gesundheit und Lebensmittelsicherheit, Veterinärstr. 2, 85764 Oberschleißheim, Deutschland

Das Gesundheitswesen ist einer der größten Wirtschaftssektoren in Deutschland. Das Statistische Bundesamt weist für das Jahr 2019 Gesundheitsausgaben in Höhe von 411 Mrd. € aus, 11,9 % des Bruttoinlandsprodukts, fast 5000 € pro Kopf. Etwa 5,75 Mio. Menschen waren im gleichen Jahr im Gesundheitswesen beschäftigt. Zum Vergleich: Im Maschinenbau, einer der wichtigsten deutschen Industriesparten, waren 257 Mrd. € Umsatz bei knapp 1,1 Mio. Beschäftigten zu verzeichnen, in der Automobilindustrie mit ihren Zulieferern knapp 1,4 Mio. Beschäftigte bei fast 440 Mrd. € Umsatz.

Hinzu kommt, dass das Gesundheitswesen durch ein konstantes und starkes Beschäftigungswachstum geprägt ist. Zwischen dem Jahr 2012 und dem Jahr 2019 hat die Zahl der Beschäftigten im Gesundheitswesen um fast 700.000 zugenommen. Von den ca. 5,75 Mio. Beschäftigten im Gesundheitswesen entfällt der größte Teil auf die Gesundheits- und Krankenpflege, etwa 880.000 Menschen sind hier tätig. In der Altenpflege waren es ca. 670.000 Menschen. Etwa 400.000 Ärztinnen und Ärzte waren 2019 der Bundesärztekammer zufolge ärztlich tätig. Insgesamt ist es ein Wirtschaftsbereich mit Hunderten von Berufen in unterschiedlichsten Berufsbildern und Tätigkeitsfeldern: von ärztlich-pflegerischen Tätigkeiten über die Biotechnologie, die industrielle Produktion von Arzneimitteln und Impfstoffen, die Medizintechnik oder Digital-Health-Bereiche bis hin zu den diversen Verwaltungstätigkeiten im Gesundheitswesen oder zur wissenschaftlichen Forschung an Hochschulen.

Der Anteil der Gesundheitsausgaben am Bruttoinlandsprodukt liegt in Deutschland in etwa auf dem gleichen Niveau wie in der Schweiz, Frankreich oder Japan. Deutlich darüber lagen die USA mit 16,8 % im Jahr 2019, deutlich darunter Länder wie Mexiko mit 5,4 %, China mit 5,2 % oder Indien mit 3,6 %. Erstrebenswert ist dabei weder die Situation in den USA, in der eine Hochleistungsmedizin für die, die es sich leisten können, mit einer Unterversorgung breiter Teile der Bevölkerung einhergeht, noch die Situation in Ländern wie Indien, wo die Gesundheitsversorgung nahezu überall unzureichend ist, nicht nur, weil Indien arm ist, sondern auch, weil es andere Prioritäten in der Verteilung der volkswirtschaftlichen Ressourcen setzt.

Während man im Allgemeinen stolz auf das Wachstum in Wirtschaftszweigen wie dem Maschinenbau oder der Automobilindustrie ist, wird Wachstum im Gesundheitswesen oft negativ interpretiert, als „Kostenexplosion“ eines volkswirtschaftlichen Konsumbereichs. Diese Wahrnehmung resultiert vor allem aus der solidarischen, nicht marktvermittelten Finanzierung der Leistungen der Sozialversicherung. Man könnte das Gesundheitswesen aber ebenso gut als investiven Bereich für das Humankapital sehen (ganz abgesehen davon, dass das Produkt „Gesundheit“ die moralische Konkurrenz um Ressourcen mit Produkten wie Autos nicht zu scheuen braucht). Hinsichtlich der Entwicklung der Gesundheitsausgaben gilt es zudem zu bedenken, dass das Gesundheitswesen ein personalintensiver Wirtschaftszweig ist, der nicht in gleicher Weise von Rationalisierungseffekten profitieren kann wie manche anderen Branchen.

Noch ein Punkt ist in diesem Zusammenhang wichtig. Das Gesundheitswesen ist – zu Recht – auf die Behandlung von Krankheiten fokussiert. Aber Gesundheit entsteht zunächst vor allem in unserem Alltag, in der Art und Weise, wie wir leben, wie wir arbeiten, wie wir uns ernähren, wie wir wohnen. Genauso wird dort unsere Gesundheit gefährdet. Nicht zufällig beginnt die vielzitierte Ottawa-Charta der WHO 1986 mit dem Hinweis darauf, dass Frieden, Wohlstand und ökologische Nachhaltigkeit elementare Voraussetzungen für die Gesundheit sind: „Grundlegende Bedingungen und konstituierende Momente von Gesundheit sind Frieden, angemessene Wohnbedingungen, Bildung, Ernährung, Einkommen, ein stabiles Öko-System, eine sorgfältige Verwendung vorhandener Naturressourcen, soziale Gerechtigkeit und Chancengleichheit. Jede Verbesserung des Gesundheitszustandes ist zwangsläufig fest an diese Grundvoraussetzungen gebunden.“

Diese Feststellung der Ottawa-Charta ist auch in der Coronakrise mit ihrer sozial ungleichen Verteilung von Infektions- und Erkrankungsrisiken wieder deutlich geworden, so wie bereits in den Jahren davor in der ungerechten Verteilung von Gesundheitschancen nach Sozialstatus. Die Lebenserwartung von Menschen der unteren Sozialstatusgruppen liegt 5–10 Jahre unter der der oberen Sozialstatusgruppen. Das dürfte übrigens auch für die Beschäftigten des Gesundheitswesens gelten. Die Entlohnung der Pflegekräfte entspricht nicht ihrer Leistung. Der erneute Angriff Russlands auf die Ukraine im Februar 2022 bringt auch einen anderen Aspekt, der in der Ottawa-Charta angesprochen wurde, in Erinnerung: Frieden ist eine elementare Voraussetzung für Gesundheit. Neben den Menschen, die durch unmittelbare Kriegseinwirkungen ums Leben kommen oder gesundheitliche Schäden davontragen, haben große Teile der Bevölkerung in der Ukraine keinen Zugang mehr zur Gesundheitsversorgung, zu einer gesunden Ernährung und sauberem Trinkwasser. Durch die Bombardierung von Industrieanlagen und Gebäuden kommt es zu einer großflächigen Umweltbelastung mit unterschiedlichen Schadstoffen, welche die Menschen noch Jahrzehnte gesundheitlich beeinträchtigen können. Zudem werden Millionen Flüchtlinge ein Leben lang, vielleicht generationenübergreifend, an den psychischen Folgen ihrer Erlebnisse zu tragen haben, in Afrika und anderen Regionen könnte die Armut wieder zunehmen, damit auch Hunger und armutsassoziierte Krankheiten, und in Europa beschneiden künftig hohe Verteidigungslasten die Spielräume der Sozial- und Gesundheitspolitik und verzögern vielleicht noch einmal überfällige Maßnahmen gegen die größte Global-Health-Herausforderung des 21. Jahrhunderts, den Klimawandel.

Auch in Deutschland zeichnen sich für die kommenden Jahre Finanzierungsengpässe und damit Verteilungskämpfe in der Gesundheitsversorgung ab. Umso mehr gilt es, die verfügbaren Ressourcen sinnvoll einzusetzen, sich dabei auch darauf zu besinnen, dass im Gesundheitswesen bei aller Notwendigkeit einer auch wirtschaftlichen Steuerung nicht wirtschaftliche Ziele oder gar Konzerngewinne im Vordergrund stehen sollten, sondern die patientenbezogene Bedarfsorientierung. Daher ist die vom Sachverständigenrat schon 2000/2001 monierte Über‑, Unter- und Fehlversorgung weiter abzubauen und – weil es nicht nur um das Gesundheitswesen geht – darüber hinaus Gesundheit in allen Politikfeldern mitzudenken.

Das vorliegende Heft des Bundesgesundheitsblatts versammelt frei eingereichte Beiträge zu unterschiedlichen Themen, von der Reduktion von SARS-CoV-2-Infektionsrisiken beim Gesundheitspersonal über den psychosozialen Versorgungsbedarf von Obdachlosen oder innovative hausärztliche Versorgungsmodelle bis hin zur präventiven Wirksamkeit von Steuererhöhungen auf den Alkoholkonsum. Solche Analysen gibt es nur, weil unser Gesundheitswesen hochgradig ausdifferenziert ist und weil es trotz aller „unmet needs“, der ungedeckten Bedarfe, finanziell doch vergleichsweise gut ausgestattet ist und über einen hochqualifizierten Beschäftigtenpool verfügt. In Versorgungsqualität übersetzt sich das in dem Maße, in dem die 5,75 Mio. Beschäftigten im Gesundheitswesen ihre vielfältigen beruflichen Kompetenzen einbringen – und indem man die Rahmenbedingungen dafür verbessert, dass sie das tun wollen und können.

